# Insights Into the Regulation of the Expression Pattern of Calvin-Benson-Bassham Cycle Enzymes in C_3_ and C_4_ Grasses

**DOI:** 10.3389/fpls.2020.570436

**Published:** 2020-10-16

**Authors:** Chidi Afamefule, Christine A. Raines

**Affiliations:** School of Life Sciences, University of Essex, Colchester, United Kingdom

**Keywords:** C_4_ photosynthesis, gene expression regulation, *cis*-regulatory elements, transcription factor binding sites, Calvin-Benson-Bassham cycle

## Abstract

C_4_ photosynthesis is characterized by the compartmentalization of the processes of atmospheric uptake of CO_2_ and its conversion into carbohydrate between mesophyll and bundle-sheath cells. As a result, most of the enzymes participating in the Calvin-Benson-Bassham (CBB) cycle, including RubisCO, are highly expressed in bundle-sheath cells. There is evidence that changes in the regulatory sequences of *RubisCO* contribute to its bundle-sheath-specific expression, however, little is known about how the spatial-expression pattern of other CBB cycle enzymes is regulated. In this study, we use a computational approach to scan for transcription factor binding sites in the regulatory regions of the genes encoding CBB cycle enzymes, SBPase, FBPase, PRK, and GAPDH-B, of C_3_ and C_4_ grasses. We identified potential *cis*-regulatory elements present in each of the genes studied here, regardless of the photosynthetic path used by the plant. The trans-acting factors that bind these elements have been validated in *A. thaliana* and might regulate the expression of the genes encoding CBB cycle enzymes. In addition, we also found C_4_-specific transcription factor binding sites in the genes encoding CBB cycle enzymes that could potentially contribute to the pathway-specific regulation of gene expression. These results provide a foundation for the functional analysis of the differences in regulation of genes encoding CBB cycle enzymes between C_3_ and C_4_ grasses.

## Introduction

C_4_ plants achieve higher photosynthetic efficiency by concentrating CO_2_ around RubisCO. In contrast with enzymes participating in C_3_ photosynthesis, C_4_-enzymes are compartmentalized to specific cell types, namely mesophyll (M) and bundle-sheath (BS) cells. Enzymes enriched in M cells include phosphoenolpyruvate carboxylase (PEPC) and pyruvate orthophosphate dikinase (Ppdk), whereas decarboxylating malic enzymes (NAD or NADP-Me) and RubisCO are enriched in the BS cells (Sheen and Bogorad, 1987; Hibberd and Covshoff, 2010; Berry et al., 2011).

During C_4_ evolution a change in localization of the enzymes involved in CO_2_ assimilation resulted in the compartmentalization of these reactions in either the M or BS cell types. A number of regulatory elements conferring a M or BS specific expression pattern have been identified in the regulatory sequences of the genes encoding PEPC, Ppdk; or NADP-ME, NAD-ME, and RubisCO (Nomura et al., 2000; Berry et al., 2011; Williams et al., 2016; Reyna-Llorens et al., 2018). To further interrogate those regulatory elements, a combination of comparative transcriptomics to identify differential expression of genes (Bräutigam et al., 2011; Aubry et al., 2014; Xu et al., 2016) and DNAse-seq to map differences in open chromatin regions between M and BS cells (Burgess et al., 2019) have been used. These studies have led to the identification of putative *cis*-regulatory elements and the trans-acting transcription factors binding to those elements, and have shown that the motifs conferring differences in expression in the C_4_ species have been recruited from pre-existing sequences in C_3_ species, rather than being generated *de novo* during the evolution of the C_4_ condition (Niklaus and Kelly, 2019).

Calvin Benson-Bassham (CBB) cycle enzymes, including RubisCO, are expressed in both C_3_ and C_4_ species. Similar to RubisCO, most of the CBB cycle enzymes are enriched in BS cells in C_4_ species (Sheen and Bogorad, 1987; John et al., 2014; Rao et al., 2016). Unlike RubisCO, little is known about the changes in the regulatory sequences of the other 10 genes encoding CCB cycle enzymes that enable such compartmentalization, limiting our ability to develop strategies to manipulate this pathway to improve photosynthetic efficiency. Here, we present a bioinformatics analysis of the regulatory sequences of genes encoding CBB cycle enzymes with the aim of identifying regulatory elements that are common to C_3_ and C_4_ species, or C_4_-specific regulatory elements that control photosynthesis and contribute to C_4_ compartmentalization. We selected four of the CBB cycle enzymes known to be redox-regulated by the ferredoxin/thioredoxin (Fd/TRX) system (Michelet et al., 2013) and that function exclusively in the CBB cycle: SBPase, FBPase (chloroplastic variant), PRK and GAPDH-B. Given the numerous independent origins of C_4_ photosynthesis that might have led to parallel evolution of *cis*-regulatory elements (Sage et al., 2012), in this paper we focus on a small subset of eight grasses from the Poaceae family whose genomes have been sequenced and annotated.

In this study we have identified putative regulatory elements that are common in both C_3_ and C_4_ species as well as C_4_-specific elements. We have also used existing data to explore the expression patterns of the trans-acting factors that have been shown or proposed to bind to these elements, suggesting a possible role in the compartmentalization of CBB cycle enzymes in C_4_ plants. The results presented here provide the basis for future functional studies.

## Materials and Methods

### DNA Sequences

Genomic sequences encoding CBB cycle enzymes of *Oryza sativa* (Ouyang et al., 2007), *Hordeum vulgare* (Beier et al., 2017; Mascher et al., 2017), *Brachypodium distachyon* (International Brachypodium Initiative, 2010), *Zea mays* (Schnable et al., 2009; Hirsch et al., 2016), *Sorghum bicolor* (McCormick et al., 2018), *Setaria viridis* (v2.1, DOE-JGI)^1^ and *Panicum virgatum* (v1.0, DOE-JGI, see footnote) were obtained from Phytozome12 (Goodstein et al., 2011). *Arabidopsis thaliana* genes (AT3G55800—*SBPase*, AT3G54050—*chlFBPase*, AT1G32060—*PRK*, and AT1G42970—*GAPDHB*) were used to identify orthologs in every species. For the genomic sequences encoding CBB cycle enzymes of *Dichanthelium oligosanthes* (Studer et al., 2016), the *A. thaliana* coding sequences were aligned against the *D. oligosanthes* genome using BLAST (Altschul et al., 1990) to find orthologous genes. Sequences used are included in Supplementary Material.

### Motif Prediction in Conserved Non-coding Sequences (CNS)

Genomic sequences were aligned using mVISTA (Frazer et al., 2004) and aligned CNSs were used as input for motif prediction using MEME (v5.1.1; Bailey et al., 2009). Motif site distribution was set to zoops and maximum motif width to the size of the shorter CNS. Predicted motifs were used as input in FIMO (Grant et al., 2011) to scan the regulatory sequences of orthologous genes in other species.

### Motif Scanning of Genomic Sequences

A collection of 529 plant transcription factor motifs validated in *A. thaliana* (O’Malley et al., 2016) were used to scan for motifs using FIMO (Grant et al., 2011) with default parameters.

### Data Processing and Visualization

Data processing and visualization were performed using R 3.6.0 (R Core Team, 2019). The dplyr package (Wickham et al., 2019) was used to filter the identified motifs by *q* < 0.05, genomic feature, and by species. The UpSetR package (Gehlenborg, 2019) was used to generate Figure 2A showing all possible interactions; and the ggplot2 (Wickham, 2016) and the gggenes packages (Wilkins, 2019) were used to generate Figure 2B.

### Transcriptomics Analysis

Transcriptomic data from RNAseq experiments in which mesophyll and bundle sheath cells were separated in *P. virgatum* (Rao et al., 2016), *S. viridis* (John et al., 2014), *Panicum hallii* (Washburn et al., 2017), and *Setaria italica* (Washburn et al., 2017) were obtained from NCBI (BioProject accession numbers: PRJNA293441, PRJEB5074, PRJNA475365). A classification-based quantification was performed using kallisto (Bray et al., 2016) with the transcriptomes and genome annotation obtained from Phytozome 12 (Goodstein et al., 2011; Bennetzen et al., 2012). In short, a kallisto index was built with the reference transcriptome of each species, and kallisto quant was used to quantify abundance of pair-end reads with default parameters. Differential expression analysis was performed with R packages DESeq (Anders and Huber, 2010) using estimateSizeFactors, estimateispersions and nbinomTest functions; DESeq2 (Love et al., 2014) using DESeq function, and edgeR (Robinson et al., 2009; McCarthy et al., 2012) using estimateCommonDisp, estimateTagwiseDisp and exactTest functions. *P*-values were adjusted with the Hochberg method in the three analyses, and only genes with adjusted *p* < 0.05 in at least one of the analyses were included in Table 1. Parallel (Tange, 2018) was used at every step to run jobs in parallel.

**TABLE 1:** Differential expression of trans-acting factors binding putative TFBS in bundle sheath and mesophyll cells of C_4_ species.

Transcription factor	*A. thaliana* name	*Panicum virgatum* name	log2 FC	*Setaria viridis* name	log2 FC	*Panicum hallii* name	log2 FC	*Setaria italica* name	log2 FC	Group
VRN1	AT3G18990	Pavir.8NG077400.2	3.6	Sevir.8G068300.1	**1.4**					cCBB
LOB	AT5G63090					Pahal.5G488600.2	3.2	Seita.5G119400.1	4.6	c34G
OBP3	AT3G55370			Sevir.3G064900.1	**6.4**	Pahal.3G092800.1	3.5	Seita.3G064100.1	**5.4**	c34P
				Sevir.3G064900.2	**6.6**	Pahal.3G092800.2	**7.9**	Seita.9G033400.1	**5.8**	
				Sevir.3G064900.3	**4.9**	Pahal.3G092800.3	**3.6**	Seita.9G452000.1	**7.2**	
				Sevir.9G032600.1	**6.5**	Pahal.9G030900.1	**5.4**			
				Sevir.9G455900.1	**7.9**	Pahal.9G513900.1	**3.1**			
				Sevir.9G455900.2	**7.0**	Pahal.9G513900.2	**9.8**			
At5g66940	At5g66940			Sevir.3G015900.1	2.2	Pahal.7G338900.1	2.8	Seita.3G014900.1	**5.1**	C3AS
AREB3	AT3G56850	Pavir.5KG593700.1	**3.3**	Sevir.9G425100.2	**5.1**					C4AP
AT3G12130	AT3G12130			Sevir.4G224600.1	–0.3	Pahal.1G071400.1	–0.9	Seita.3G029300.2	–0.9	C3AP
				Sevir.4G224600.2	–1.7	Pahal.1G071400.3	–7.3	Seita.4G214800.1	–0.5	
ERF5	AT5G47230			Sevir.1G261900.1	–1.1	Pahal.9G383200.1	–**3.0**	Seita.1G257600.1	–**1.7**	cSFG
AS2	AT1G65620			Sevir.3G246200.2	–5.4			Seita.5G408700.1	–**3.4**	c34G
					–3.4					
ERF1	AT3G23240			Sevir.8G100900.1	–**5.3**	Pahal.2G139200.1	–**3.4**	Seita.2G138400.1	–**2.9**	c34G
				Sevir.9G504700.1	–**3.7**	Pahal.8G262800.1	1.9	Seita.9G500100.1	–1.8	
ERF9	AT5G44210	Pavir.5NG539500.1	–2.4	Sevir.3G196300.1	1.2			Seita.5G348000.1	–**1.4**	c34G
				Sevir.5G352700.1	–**2.5**					
ERF15	AT2G31230			Sevir.2G118100.1	–**4.5**	Pahal.8G107700.1	–1.5	Seita.2G112200.1	–**2.1**	c34G
				Sevir.8G182200.1	5.7			Seita.8G173100.1	**7.6**	
								Seita.8G237900.1	**4.3**	
TCX2	AT4G14770			Sevir.2G055300.3	–6.2	Pahal.3G490800.1	0.7	Seita.2G050700.1	–**3.6**	C3AF
				Sevir.3G398500.1	0.7	Pahal.9G158300.1	**7.0**	Seita.3G382000.1	–0.7	
				Sevir.9G159400.4	**5.0**			Seita.9G161100.3	5.1	
				Sevir.9G159400.6	**4.8**			Seita.9G161100.2	7.5	
				Sevir.9G159400.1	2.7					
				Sevir.9G159400.7	**4.1**					
ERF73	AT1G72360	Pavir.9NG798900.1	5.5	Sevir.2G400300.5	**2.9**	Pahal.2G447100.1	**1.8**	Seita.2G390000.1	**3.6**	cSFG
				Sevir.9G520900.1	–**1.4**	Pahal.2G447100.2	**2.2**	Seita.2G390000.3	**1.2**	
				Sevir.9G521200.2	–**4.1**	Pahal.9G580000.1	–**1.4**	Seita.9G516500.1	–**1.9**	
						Pahal.9G580100.1	–**2.1**	Seita.9G516600.1	3.0	
						Pahal.9G580200.1	–**2.1**	Seita.9G516700.1	–**4.2**	
						Pahal.9G580200.2	–**2.1**	Seita.9G516700.2	–**4.1**	
								Seita.9G516800.1	–**3.6**	
								Seita.9G516800.2	**3.4**	
bZIP16	AT2G35530			Sevir.2G084800.1	0.8	Pahal.1G023000.4	**1.1**	Seita.3G090500.2	**1.2**	C4AP
				Sevir.3G092500.1	–0.7	Pahal.1G023000.5	–0.7	Seita.3G090500.3	–9.7	
						Pahal.1G023000.6	**1.6**	Seita.9G474400.1	0.3	
						Pahal.3G063900.2	0.6			
						Pahal.9G536900.1	0.5			

**The expression level of each Arabidopsis thaliana transcription factor ortholog was obtained from RNAseq data of Panicum virgatum, Setaria viridis, Panicum hallii and Setaria italica. Every A. thaliana transcription factor had one or more orthologs in the target species. The log2 fold change between bundle sheath and mesophyll cells was calculated using three different methods to evaluate the difference in expression level between cell types (see “Materials and Methods” section), and only genes with adjusted p < 0.05 in at least one of the methods were included. log2 fold values in bold indicate that they are significant using the three methods. Transcription factors were classified as putative activators (in green) if they were enriched in bundle sheath cells, or putative repressors (in gold) if they were enriched in mesophyll cells. Transcription factors in blue showed enrichment in both bundle sheath and mesophyll cells among different orthologs. Last column indicates the group to which each transcription factor belongs (see Figure 2): common_CBB (cCBB), common_SFG (cSFG), common_GAPDHB (c34G), common_PRK (c34P), C3-Absent_SBPase (C3AS), C3-Absent_PRK (C3AP), and C4-Absent_PRK (C4AP).**

To construct Supplementary Table 1, we used the 57 *A. thaliana* transcription factors that have been shown to bind the identified transcription factor binding sites (TFBS, 50 shared by different orthologous genes, Figure 2; plus 7 absent from C_3_ or C_4_ species, Supplementary Figure S5). We identify the orthologous genes in grass species and evaluate their enrichment in M or BS cells using publicly available transcriptomic data for *S. viridis* (John et al., 2014), *S. italica* (Washburn et al., 2017), *P. virgatum* (Rao et al., 2016), *P. halli* (Washburn et al., 2017), *Z. mays* (Chang et al., 2012), and *S. bicolor* (Döring et al., 2016). All these databases separate M and BS cells from whole leaves. We identified 10 orthologous genes significantly enriched (adj. *p* < 0.05) in *P. virgatum*, which corresponded to 8 genes in *A. thaliana*. For *P. halli* we identified 21 orthologs corresponding to 11 *A. thaliana* genes. For *S. viridis* we identified 53 orthologs corresponding to 26 *A. thaliana* genes. For *S. italica* we identified 46 orthologs corresponding to 22 *A. thaliana* genes. For *Z. mays* we identified 10 orthologs corresponding to 4 *A. thaliana* genes. For *S. bicolor* we identified 2 orthologs corresponding to 2 *A. thaliana* genes. In Table 1, we only included the *A. thaliana* genes for which the log2 fold was at least 1, and with consistent data from at least two species. We also removed *Z. mays* and *S. bicolor* orthologous genes as their transcriptomic data did not add any information on the *A. thaliana* genes included on Table 1.

## Results

**FIGURE 1:**
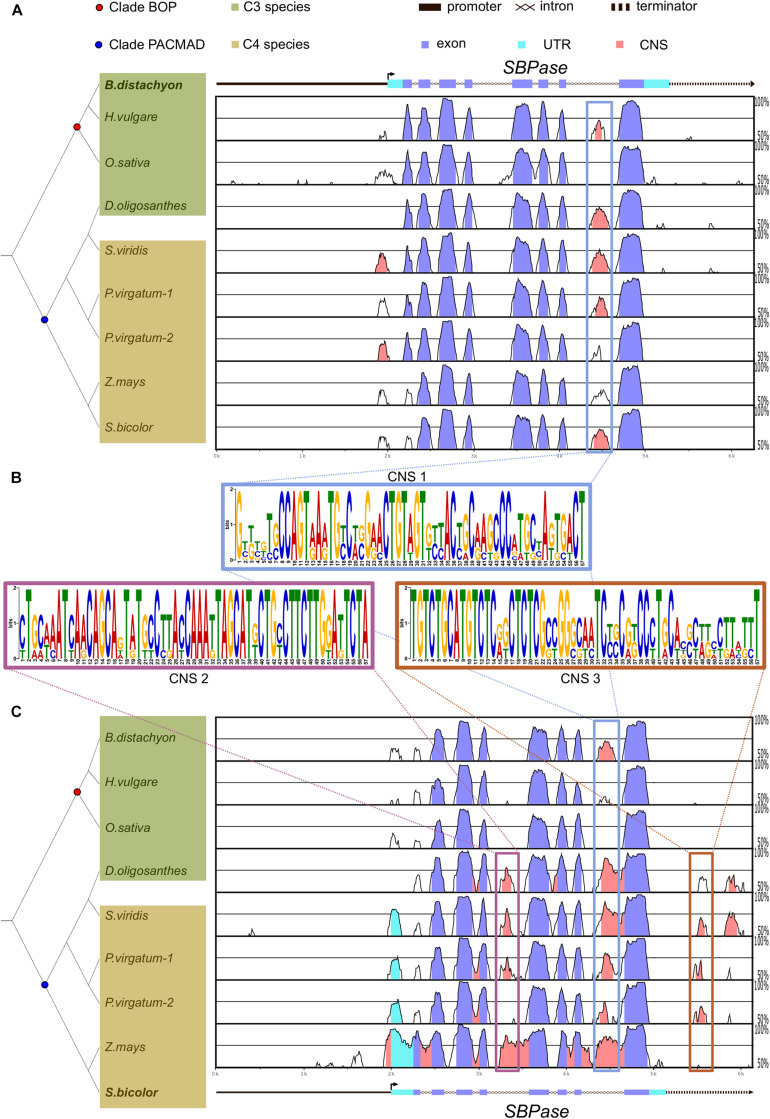
*SBPase* coding sequence is highly conserved among C_3_ and C_4_ grasses in comparison to putative regulatory regions. **(A,C)** mVISTA plots of *Brachypodium distachyon* **(A)** and *Sorghum bicolor* **(C)** *SBPase* aligned to *SBPase* orthologs in C_3_ and C_4_ grasses. Genomic region includes approximately 2 kb upstream from the transcription start site and 1 kb after the end of the 3′ untranslated region (UTR). UTRs, exons, and introns are annotated. The vertical line with a small perpendicular arrow indicates the transcription start site and the arrowhead the orientation of the gene. The graph shows sequences with 50–100% identity and regions with > 70% identity within 50 base pairs are highlighted in purple if they are located in exons, in cyan if they are located in UTRs, or in pink if they are located outside exons or UTRs. Boxes highlight conserved non-coding sequences (CNSs), and the predicted position weight matrix for each conserved sequence is included **(B)**. On the left side, the phylogenetic relationship between C_3_ (in green) and C_4_ (in brown) grasses is shown. Common ancestor of BOP clade and PACMAD clade species are shown as a red and as a blue dot, respectively. Note that *Dichanthelium oligosanthes* is a C_3_ species within the PACMAD.

To account for the numerous independent origins of C_4_ photosynthesis, we focus on a small subset of eight grasses: *Oryza sativa*, *Hordeum vulgare*, *Brachypodium distachyon*, *Dichanthelium oligosanthes*, *Zea mays*, *Sorghum bicolor*, *Panicum virgatum*, and *Setaria viridis*. All of these plant species belong to the Poaceae family and shared a common ancestor around 50 million years ago. *O. sativa*, *H. vulgare*, *B. distachyon*, and *D. oligosanthes* perform C_3_ photosynthesis, whereas *Z. mays, S. bicolor, P. virgatum*, and *S. viridis* perform C_4_ photosynthesis. Notably, *D. oligosanthes* belongs to the PACMAD clade (Figure 1), to which all selected C_4_ species belong, and shares a common ancestor with them around 15 million years ago (Studer et al., 2016). To identify conserved regulatory regions in genes encoding CBB cycle enzymes of C_3_ and C_4_ grasses, we aligned each gene against its orthologous gene in a representative C_3_ species (*B. distachyon*; Figure 1A and Supplementary Figures S1A, S2A, S3A) and against its orthologous gene in a representative C_4_ species (*S. bicolor*; Figure 1C and Supplementary Figures S1C, S2C, S3B). The genomic sequence including potential promoters [2000 base pair (bp)] upstream from the annotated transcription start site (or start codon otherwise) and potential terminators (1,000 bp downstream from the end of 3′UTR or stop codon) was used to allow for the identification of putative regulatory regions outside coding sequences. Regions showing between 50 and 100% identity were plotted and conserved regions with over 70% identity were colored depending on the genomic feature (Figures 1A,C; coding sequences in purple, untranslated regions [UTRs] in cyan, and intergenic regions and introns in pink) As expected, most of the coding sequences were conserved among all orthologous genes, whereas only parts of the introns and intergenic sequences showed over 70% identity. We defined those regions as conserved non-coding sequences (CNS). For *SBPase*, we identified one CNS located at the last intron of most orthologs (Figures 1A,C), and two CNSs found only in *SBPase* orthologous genes from PACMAD species (C_4_ species + *D. oligosanthes*; Figure 1C). In addition, we found one CNS located at the 5′ intergenic region of all *PRK* genes (Supplementary Figure S1), and two CNSs located at the 5′ intergenic region of *FBPase* genes from PACMAD species (Supplementary Figure S2). To further characterize these CNSs, they were subjected to motif prediction using MEME (Bailey et al., 2009), which generated a position weight matrix for the predicted motifs (Figure 1B and Supplementary Figures S1B, S2B). We used these motifs to scan the orthologous genes of other species, and identified the *PRK* CNS in the intergenic regions of *PRK* orthologs in non-grasses species (Supplementary Dataset 1). These results indicate that there are conserved potential *cis*-regulatory sequences shared between C_3_ and C_4_ species. However, this alignment approach is based on sequence identity over at least 50 bp; so it was possible that smaller motifs, such as transcription factor binding sites (TFBS) could have been disregarded.

**FIGURE 2:**
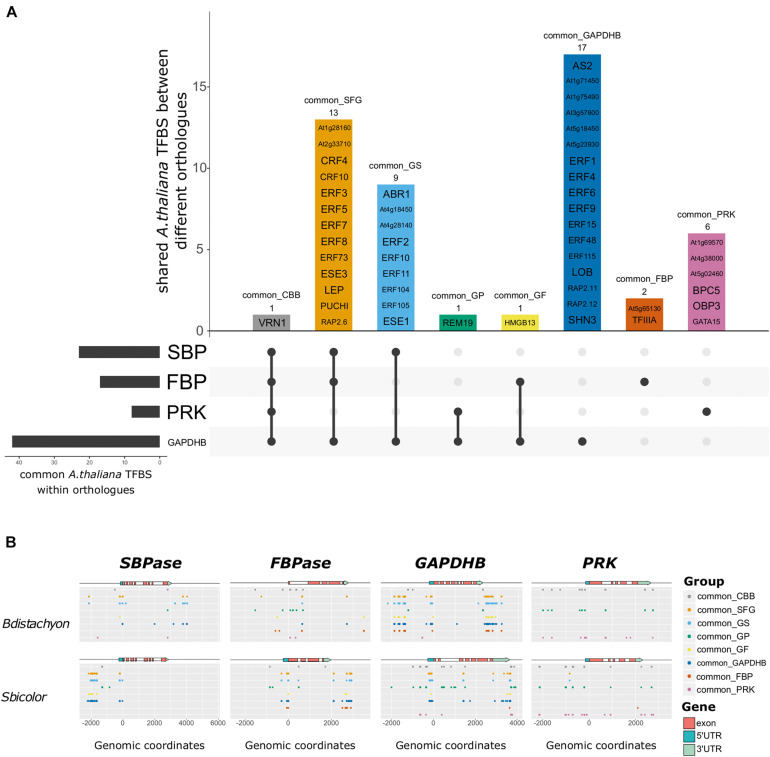
*Arabidopsis thaliana* transcription factor binding sites (TFBS) identified in the potential regulatory sequences of genes encoding C_3_ and C_4_ Calvin-Benson-Bassham (CBB) cycle enzymes. **(A)** Upset plot showing the identified *A. thaliana* TFBS and in which orthologous genes they are found. Horizontal bars represent the number of common motifs identified within orthologs, vertical bars represent the motifs shared between different orthologous genes, as indicated by the dots below. The name of the *A. thaliana* TFBS is included inside the vertical bars, and the number of motifs as well as the name of the gene group are indicated above. There is only one common motif shared across all orthologs (common_CBB: VRN1, gray bar), and most of the common motifs identified in *GAPDHB* are not common in the genes encoding other enzymes (common_GADPHB). In addition, many motifs are shared between *GAPDHB*, *FBPase*, and *SBPase* (common_SFG), whereas most *PRK* motifs are not shared with the genes encoding other enzymes (common_PRK). **(B)** Localization of each gene group in the genomic region around genes encoding CBB cycle enzymes in *Brachypodium distachyon* and *Sorghum bicolor*. The *x*-axis corresponds to the genomic coordinates with the start codon corresponding to the + 1 position. The colored arrow represents the gene structure with UTRs in blue, exons in red, and potential promoter and terminator as a black line. The dots represent the genomic coordinates of each of the motifs within each gene group. Different gene groups are separated along the *y*-axis. Despite being comprise by the same TFBS, the distribution of the dots changes between species. Notably, TFBS can be found at multiple coordinates in the same gene. Most of the trans-acting factors binding to the identified *A. thaliana* TFBS belong to the same family, and often bind to similar sequences. In fact, TFBS tend to cluster in discrete regions that might play a role in the regulation of the expression of the corresponding gene.

To evaluate the presence of TFBS in the regulatory regions of genes encoding CBB cycle enzymes, a dataset containing validated TFBS in *Arabidopsis thaliana* (O’Malley et al., 2016) was used to scan the putative regulatory sequences (intergenic regions, untranslated regions, and introns) of orthologous genes, i.e., *SBPase* orthologs across the subset of eight grass species were scanned at the same time. We first determined the *A. thaliana* TFBS shared between orthologous genes, and used those to compare between the genes encoding the selected four CBB cycle enzymes (Figure 2A). This way, we identified one TFBS present in all of the potential regulatory sequences (common_CBB, in Figure 2A) that was bound by VRN1 in *A. thaliana*. This TFBS was also identified it in the putative regulatory regions of genes encoding photorespiratory (*GDCH*) and housekeeping proteins (*CBP20*) (Supplementary Dataset 2), suggesting that it might play a regulatory role not limited to photosynthetic genes. We also identified 13 putative TFBS shared between *SBPase, FBPase*, and *GAPDHB* orthologous genes (common_SFG), 9 TFBS shared between *GAPDHB* and *SBPase* orthologous genes (common_GS), one shared between *GAPDHB* and *PRK* orthologous genes (common_GP), and one TFBS shared between *GAPDHB* and *FBPase* orthologous genes (common_GF). In addition, 17, 2, and 6 putative TFBS were shared between *GAPDHB* orthologs (common_GAPDHB), *FBPase* orthologs (common_FBP), and *PRK* orthologs (common_PRK); but not between any other group of orthologous genes. Notably, these common sequences can be found in potential regulatory sequences of other orthologous genes from some but not in all of the species in the study. The fact that all the *A. thaliana* TFBS found in *SBPase* orthologs are shared with other genes (common_SFG, common_GS) whereas most of the TFBS found in *PRK* orthologs are not shared (common_PRK) suggests different mechanisms for the regulation of the expression of the genes encoding CBB cycle enzymes. Most of the trans-acting factors binding to the identified TFBS belong to the Apetala2/Ethylene-Response-Factor (AP2/ERF) family, and often recognize similar binding sites. A comparison between the location of the *A. thaliana* TFBS at the putative regulatory regions of the orthologs in the selected species (Figure 2B and Supplementary Figure S4) revealed that the TFBS tend to cluster together in discrete regions of the putative regulatory sequences, although the genomic coordinates of these clusters change between species.

We used a similar approach to identify C_4_-specific TFBS contributing to the difference in expression pattern between C_3_ and C_4_ species. After scanning together orthologous genes (i.e., all *SBPase* orthologous genes with the *A. thaliana* validated TFBS), we selected TFBS absent from the putative regulatory regions of genes encoding C_3_-enzymes (Supplementary Figure S5). Using this approach (choosing absent motifs from genes encoding C_3_ enzymes rather than present motifs in all genes encoding C_4_ enzymes), it was possible to account for the multiple independent origins of C_4_ photosynthesis. Three TFBS were found absent from *SBPase* C_3_-genes, bound by At5g66940, BZR1, and CEJ1 in *A. thaliana*; one absent from *FBPase* C_3_-genes (bound by TCX2) and one absent from *PRK* C_3_-genes (bound by At3g12130). In addition, using the same approach to identify TFBS absent from genes encoding C_4_ enzymes revealed two C_3_-specific motifs in the 5′ intergenic region of *PRK* (bound by AREB3 and bZIP16). These results suggest that there are C_3_- and C_4_-specific TFBS that might contribute to the compartmentalization of C_4_ CBB cycle enzymes.

To further understand how the identified TFBS might regulate the expression pattern of CBB cycle enzymes, we obtained the transcriptomic data from a collection of RNA-seq experiments on C_4_ species where samples were taken separately from mesophyll and bundle sheath cells (John et al., 2014; Rao et al., 2016; Washburn et al., 2017), and assessed the expression pattern of the trans-acting factors. Despite the complexities of using data from different experiments, and the limited validation of the interaction between trans-acting factors and the identified TFBS (i.e., only validated in the C_3_ species *A. thaliana*), we identified ten trans-acting factors differentially enriched in M and BS cell types (Table 1). These trans-acting factors were the orthologs of the validated trans-acting factors in *A. thaliana*, and could be classified into three categories in regard to BS-specific enrichment (Table 1): (1) putative activators, if all the orthologs were consistently enriched in BS over M cells, such as the orthologs of *At5g66940*, whose TFBS are only found in *SBPase* orthologs of C_4_ species; (2) putative repressors, if all the orthologs were consistently enriched in M over BS cells, for example the orthologs of *At3g12130*, whose TFBS are only found in *PRK* orthologs of C_4_ species; (3) broad regulators, if enrichment of orthologous genes was inconsistent within species (some were enriched in BS over M cells while others were enriched in M over BS cells), such as the orthologs of *TCX2*, which are found enriched in both M and BS cells, and whose TFBS are only found in *FBPase* orthologs of C_4_ species.

## Discussion

In this study, we have used publicly available data to analyze putative regulatory regions of genes encoding a selected subset of CBB cycle enzymes (SBPase, FBPase, PRK, and GAPDHB) in C_3_ and C_4_ species. We used two different approaches to identify potential regulatory elements that might contribute to the compartmentalization of CBB enzymes in C_4_ species. The alignment of the genomic regions of the orthologs encoding the selected CBB cycle enzymes allowed us to identify conserved non-coding sequences (CNSs) shared by C_3_ and C_4_ orthologous genes, whereas the scanning of putative regulatory regions with TFBS validated in *A. thaliana*, allowed us to identify putative C_4_-specific regulatory elements. The results presented here provide new information on putative regulatory elements of the genes encoding SBPase, FBPase, PRK, and GAPDHB in both C_3_ and C_4_ species and although we do not provide experimental evidence in this paper the results form the basis for future functional studies.

The alignment of the genomic regions of orthologous CBB genes revealed a number of CNSs shared between C_3_ and C_4_ species. We identified a highly conserved sequence in the 5′ intergenic region of every *PRK* gene (Supplementary Figure S1). This CNS stands out because of its length (113 bp) and the level of conservation, as it can be found in C_3_ species even outside of Poaceae (Supplementary Dataset S1). These attributes suggest that this region could have contributed to the regulation of *PRK* expression throughout evolutionary history. In contrast, the CNS identified in the last intron of *SBPase* orthologous genes (Figure 1) was only found in species belonging to Poaceae, suggesting that the possible contribution to the regulation of *SBPase* genes is limited to Poaceae species (Supplementary Dataset S1). Nevertheless, the location of this conserved region highlights the relevance of searching for regulatory elements outside of the up- and down-stream non-coding sequences of genes (Rose, 2019). Additionally, we also identified CNSs conserved only within the more closely related species of the PACMAD clade (*D. oligosanthes*, *Z. mays, S. bicolor, P. virgatum*, and *S. viridis*) but not within the more distant related species of the BOP clade (*O. sativa*, *H. vulgare*, and *B. distachyon*; Figure 1 and Supplementary Figure S2). The fact that these CNSs are only shared between the species of the PACMAD clade, including *D. oligosanthes* which performs C_3_ photosynthesis, suggests that these CNSs do not play a role in C_4_ compartmentalization and instead they are a result of shared evolutionary history. However, the significance of the contribution of these conserved regions to the levels or patterns of expression of these genes remains to be elucidated experimentally.

Using a different approach based on validated TFBS and their trans-acting factors in *A. thaliana*, we identified putative (i.e., non-validated in grasses) TFBS shared by the genes encoding CBB cycle enzymes in both C_3_ and C_4_ species (Figure 2A), as well as C_4_-specific (C_3_-absent) putative TFBS (Supplementary Figure S5). We found three putative TFBS absent from *SBPase* C_3_-genes, one absent from *FBPase* C_3_-genes, and one absent from *PRK* C_3_-genes. The identification of *A. thaliana* TFBS in genes encoding C_4_ CBB cycle enzymes supports the hypothesis that C_4_ genes co-opted regulatory elements of C_3_ genes to establish their restricted expression pattern (Brown et al., 2011; Xu et al., 2016; Borba et al., 2018; Reyna-Llorens et al., 2018). Notably, we did not identify any C_3_- or C_4_-specific putative TFBS in the regulatory regions of *GAPDHB* orthologs (Supplementary Figure S5) but found more shared TFBS between C_3_ and C_4_ *GAPDHB* orthologs (Figure 2A). Despite being expressed in BS cells, which should allow for CBB cycle function in those cells, *GAPDHB* is enriched in M cells (Majeran et al., 2005; Rao et al., 2016). The lack of C_4_-specific putative TFBS in *GAPDHB* regulatory regions suggests that its expression might be regulated similarly in both C_3_ and C_4_ plants. Most of the identified TFBS are recognized by members of the AP2/ERF family in *A. thaliana*, which supports the results of a recent study in which this family of TFBS was enriched in the regulatory regions of C_4_ photosynthetic genes (Burgess et al., 2019). We realized that these putative TFBS were often quite similar and cluster together at specific locations in the genome and this warrants further investigation to explore the functional significance. Despite the similarities, we only identified one TFBS, bound by VRN1 in *A. thaliana*, in the putative regulatory regions of every gene selected for this study, but its presence in other non-photosynthetic genes indicates that VRN1 is unlikely to be exclusive to the regulation of the expression of genes encoding CBB cycle enzymes. Furthermore, the variety in the putative TFBS identified in different sets of orthologous genes indicates differences in the regulatory networks controlling their expression. These results suggest that there is no “master” transcriptional regulator coordinating the expression of the genes encoding CBB cycle enzymes, in contrast to what has been reported in other metabolic pathways (Okada et al., 2009; Nützmann et al., 2018). In addition, the lack of a unique, “master” regulator would emphasize the importance of the simultaneous manipulation of multiple targets to increase CBB cycle efficiency (Simkin et al., 2015, 2017; López-Calcagno et al., 2020).

Based on data validated in the model plant *A. thaliana*, we used a computational approach to identify *cis*-regulatory elements whose putative trans-acting factors might play a role in C_4_ compartmentalization. These data have been used to investigate the putative role of orthologous genes in other crops (Capote et al., 2018; Moon et al., 2018; Burgess et al., 2019; Zeng et al., 2019; DeMers et al., 2020; Elzanati et al., 2020; Gray et al., 2020; Zhou et al., 2020), and allow us to generate a compelling hypothesis, as it is expected that similar DNA-binding domains of trans-acting factors would have similar DNA sequence preferences (Lambert et al., 2019). However, several complementary experimental approaches will be needed to provide evidence of functional significance in C_4_ plants. To confirm the TFBS in different species, transcription factor binding assays such as DAP-seq (O’Malley et al., 2016) could be developed in some of the grass species examined in this study. To assess the chromatin accessibility of potential regulatory regions, experiments such as DNAse-seq (Zhang and Jiang, 2015) or ATAC-seq (Buenrostro et al., 2015; Bajic et al., 2018; Maher et al., 2018), could be implemented. To enhance our ability to detect regulatory elements within coding sequences (Reyna-Llorens et al., 2018), functional assays that discriminate between conserved sites with a regulatory role and conserved sites with a coding sequence role could be developed. Finally, the generation of transcriptomic data from different species using a comparable sampling process, should allow us to unveil consistent pattern of expression among different species.

Taking all of our results together, we propose that the compartmentalization of the CBB cycle enzymes investigated in this study has occurred through the recruitment of TFBS whose trans-acting factors are enriched in either one of the C_4_ cell types. The expression pattern of any gene is determined by a combination of the TFBS present and the corresponding trans-acting factors binding to these regulatory regions at any given time. It then follows that the expression pattern of any gene can be changed either by recruiting new TFBS or by altering the expression pattern of the trans-acting factors. Thus, to enrich the expression of C_4_ enzymes in BS cells, new TFBS could be recruited into gene regulatory regions of C_4_ species to confer BS-specific expression. Alternatively, trans-acting factors could become enriched in BS cells to promote the expression of C_4_ enzymes in BS cells (as the predicted putative activators), or these factors could become enriched in M cells to repress the expression of C_4_ enzymes in M cells (predicted putative repressors). This transcriptional regulation would likely be complemented by regulation at post-transcriptional and/or post-translational level to achieve a precise regulation of the expression pattern of CBB cycle enzymes.

To our knowledge, and excluding the extensive work on *RubisCO* (discussed in Hibberd and Covshoff, 2010; Berry et al., 2011; Schlüter and Weber, 2020), this is the first study to focus specifically on the differences in the regulatory sequences of CBB cycle genes between C_3_ and C_4_ species. These results provide a hypothetical foundation for future functional analysis. Future experiments should include the *in vivo* validation of the trans-acting factors binding to *cis*-regulatory elements, and the resultant regulation of CBB cycle genes; the transfer of C_4_-specific transcription factors into C_3_ species to establish a C_4_-like expression pattern; or the precise genome editing of the *cis*-elements to evaluate their contribution to compartmentalization in C_4_ plants.

## Data Availability Statement

All datasets presented in this study are included in the article/Supplementary Material.

## Author Contributions

CAR conceived the study and supervised the research with input from CA. CA performed the analysis and wrote the manuscript with input from CAR. All authors contributed to the article and approved the submitted version.

## Conflict of Interest

The authors declare that the research was conducted in the absence of any commercial or financial relationships that could be construed as a potential conflict of interest.

## Footnotes

1 http://phytozome.jgi.doe.gov/
