# Supplementary material for: Insights Into the Regulation of the Expression Pattern of Calvin-Benson-Bassham Cycle Enzymes in C3 and C4 Grasses
Source: Front Plant Sci. 2020 Oct 16;11:570436. doi: 10.3389/fpls.2020.570436 (PMC7595957; doi:10.3389/fpls.2020.570436)
Supplement: Supplementary file 2 [file Data_Sheet_2.PDF]

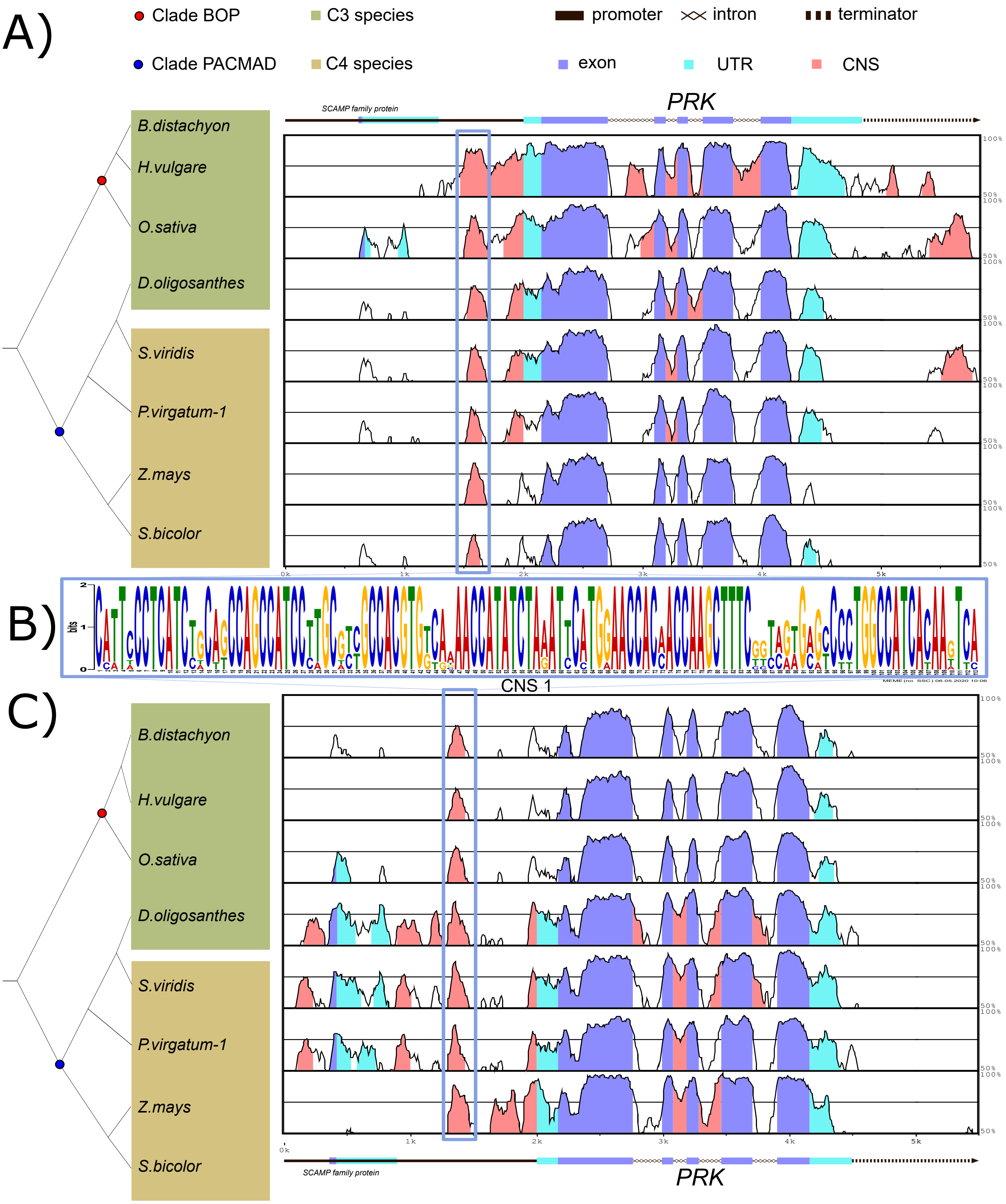

Supplementary Figure S1.- *PRK* coding sequence is highly conserved among C_3 and C_4 grasses in comparison to putative regulatory regions. (A, C) mVISTA plots of *Brachypodium distachyon* (A) and *Sorghum bicolor* (C) *PRK* aligned to *PRK* orthologues in C_3 and C_4 grasses. Genomic region includes approximately 2kb upstream from the transcription start site and 1kb after the end of the 3' untranslated region (UTR). UTRs, exons, and introns are annotated. The arrowhead indicates the orientation of the gene. A second gene (*SCAMP family protein*) is found upstream *PRK* coding sequence in both *Brachypodium distachyon* and *Sorghum bicolor*, resulting in conservation peaks associated to a coding sequence and UTRs. Boxes highlight conserved non-coding sequences (CNSs), and the predicted position weight matrix for each conserved sequence is included (B). On the left side, phylogenetic relationship between C_3 (in green) and C_4 (in brown) grasses. Common ancestor of BOP clade and PACMAD clade species are shown as a red and as a blue dot, respectively. A common CNS across all species was identified. The predicted motif is 113bp long and found also in other C_3 and C_4 species (Supplementary Dataset 1)
