# Supplementary material for: Insights Into the Regulation of the Expression Pattern of Calvin-Benson-Bassham Cycle Enzymes in C3 and C4 Grasses
Source: Front Plant Sci. 2020 Oct 16;11:570436. doi: 10.3389/fpls.2020.570436 (PMC7595957; doi:10.3389/fpls.2020.570436)
Supplement: Supplementary file 3 [file Data_Sheet_3.PDF]

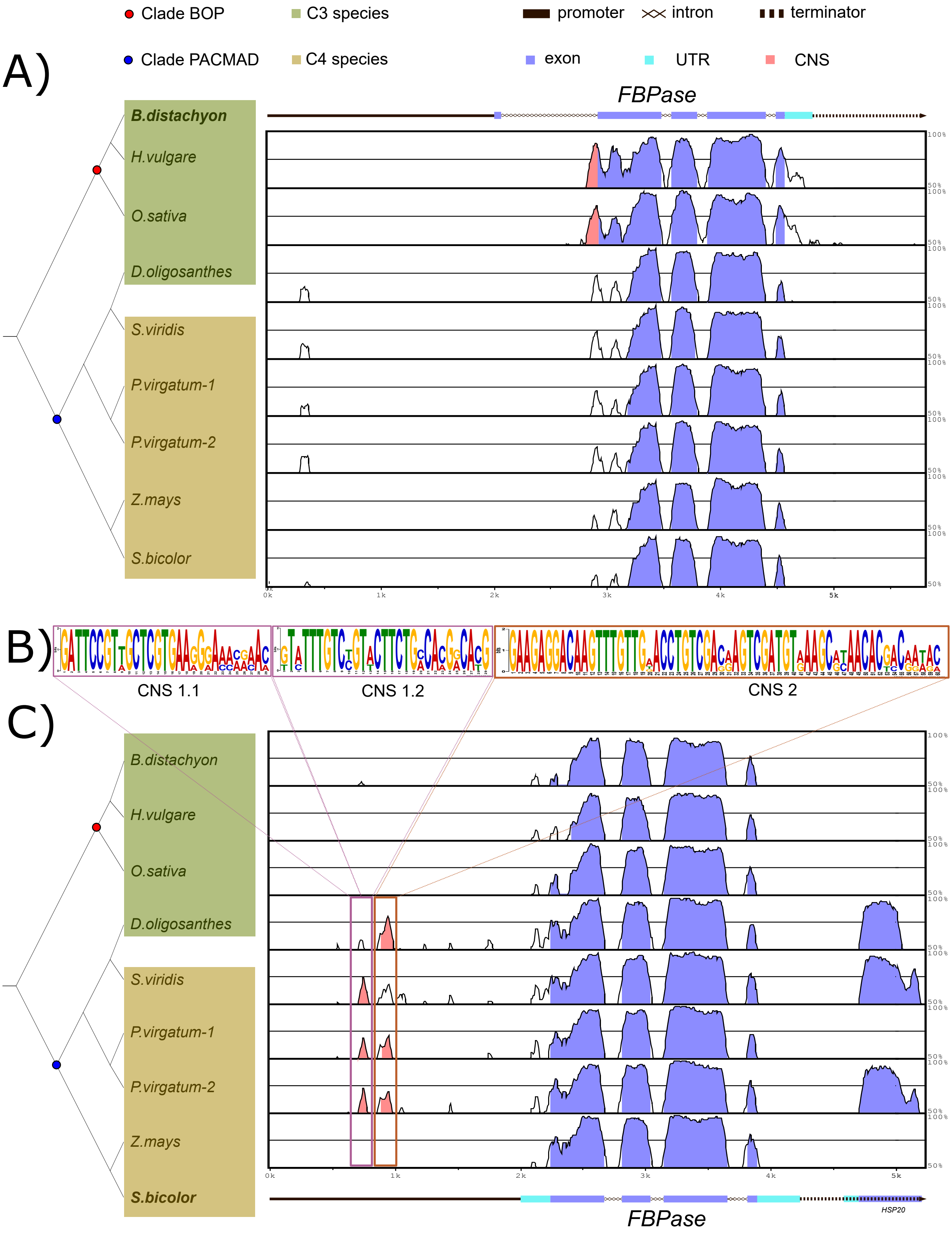

Supplemetary Figure S2.- *FBPase* coding sequence is highly conserved among C$_3$ and C$_4$ grasses in comparison to putative regulatory regions. (A, C) mVISTA plot of *Brachypodium distachyon* (A) and *Sorghum bicolor* (C) *FBPase* aligned to *FBPase* orthologues in C$_3$ and C$_4$ grasses. Genomic region includes approximately 2kb upstream from the transcription start site and 1kb after the end of the 3' untranslated region (UTR). UTRs, exons, and introns are annotated. The arrowhead indicates the orientation of the gene. A second gene (*HSP20*) is found downstream *FBPase* coding sequence in *Sorghum bicolor*, resulting in conservation peaks associated to a coding sequence. Boxes highlight conserved non-coding sequences (CNSs), and the predicted position weight matrix for each conserved sequence is included (B). On the left side, phylogenetic relationship between C$_3$ (in green) and C$_4$ (in brown) grasses. Common ancestor of BOP clade and PACMAD clade species are shown as a red and as a blue dot, respectively. CNSs were identified between species belonging to the PACMAD clade and one of the CNS was hosting two adjacent motifs (CNS1.1 and CNS1.2 in B).
