# Supplementary material for: Insights Into the Regulation of the Expression Pattern of Calvin-Benson-Bassham Cycle Enzymes in C3 and C4 Grasses
Source: Front Plant Sci. 2020 Oct 16;11:570436. doi: 10.3389/fpls.2020.570436 (PMC7595957; doi:10.3389/fpls.2020.570436)
Supplement: Supplementary file 4 [file Data_Sheet_4.PDF]

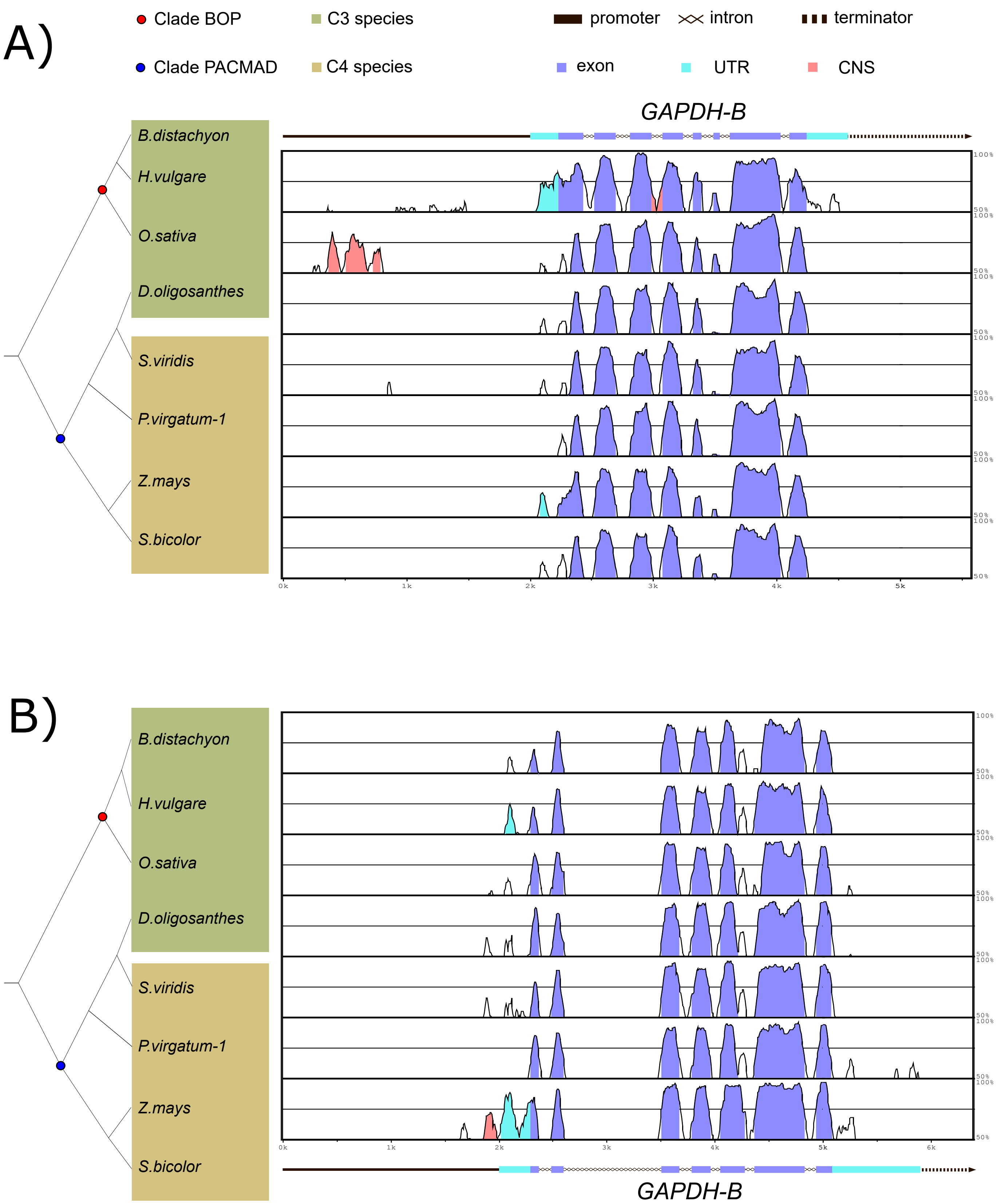

Supplementary Figure S3.- *GAPDHB* coding sequence is highly conserved among C_3 and C_4 grasses in comparison to putative regulatory regions. (A, B) mVISTA plot of *Brachypodium distachyon* (A) and *Sorghum bicolor* (B) *GAPDHB* aligned to *GAPDHB* orthologues in C_3 and C_4 grasses. Genomic region includes approximately 2kb upstream from the transcription start site and 1kb after the end of the 3' untranslated region (UTR). UTRs, exons, and introns are annotated. The arrowhead indicates the orientation of the gene. No conserved non-coding sequences (CNS) were detected between C_3 or C_4 orthologues. On the left side, phylogenetic relationship between C_3 (in green) and C_4 (in brown) grasses. Common ancestor of BOP clade and PACMAD clade species are shown as a red and as a blue dot, respectively.
