# Supplementary material for: Insights Into the Regulation of the Expression Pattern of Calvin-Benson-Bassham Cycle Enzymes in C3 and C4 Grasses
Source: Front Plant Sci. 2020 Oct 16;11:570436. doi: 10.3389/fpls.2020.570436 (PMC7595957; doi:10.3389/fpls.2020.570436)
Supplement: Supplementary file 5 [file Data_Sheet_5.PDF]

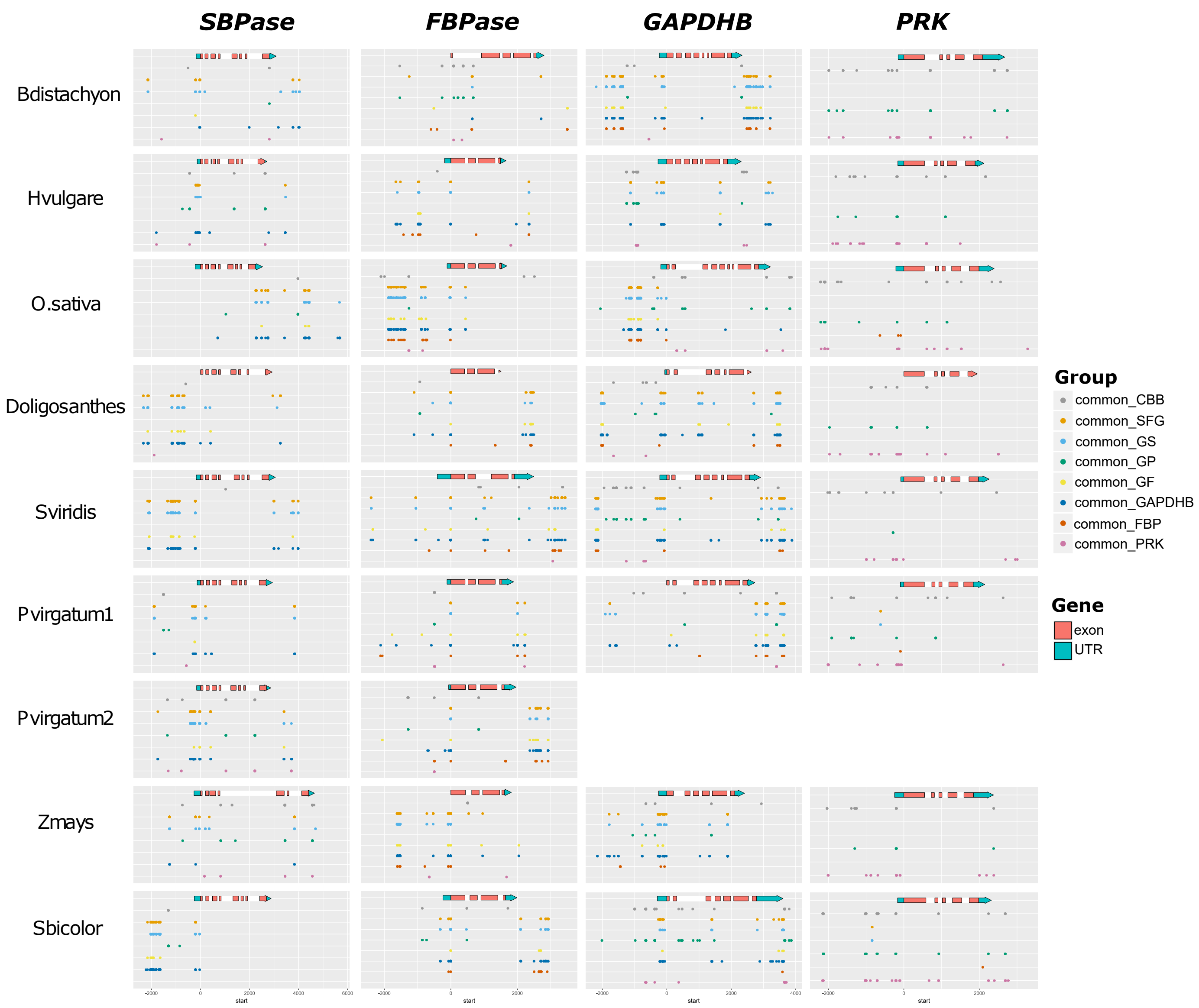

Suplementary Figure S4.- Localization of *Arabidopsis thaliana* transcription factor binding sites identified in the potential regulatory regions of genes encoding C_3 and C_4 Calvin-Benson-Bassham cycle enzymes in the selected species for this study. The dots represent the genomic coordinates of each of the motifs within each gene group (see text and Figure 2 for group description). The x-axis corresponds to the genomic coordinates with the start codon corresponding to the +1 position. The colored arrow represents the gene structure with untranslated regions (UTRs) in blue and exons in red. Different gene groups are separated along the y-axis.
