# Supplementary material for: Insights Into the Regulation of the Expression Pattern of Calvin-Benson-Bassham Cycle Enzymes in C3 and C4 Grasses
Source: Front Plant Sci. 2020 Oct 16;11:570436. doi: 10.3389/fpls.2020.570436 (PMC7595957; doi:10.3389/fpls.2020.570436)
Supplement: Supplementary file 6 [file Data_Sheet_6.PDF]

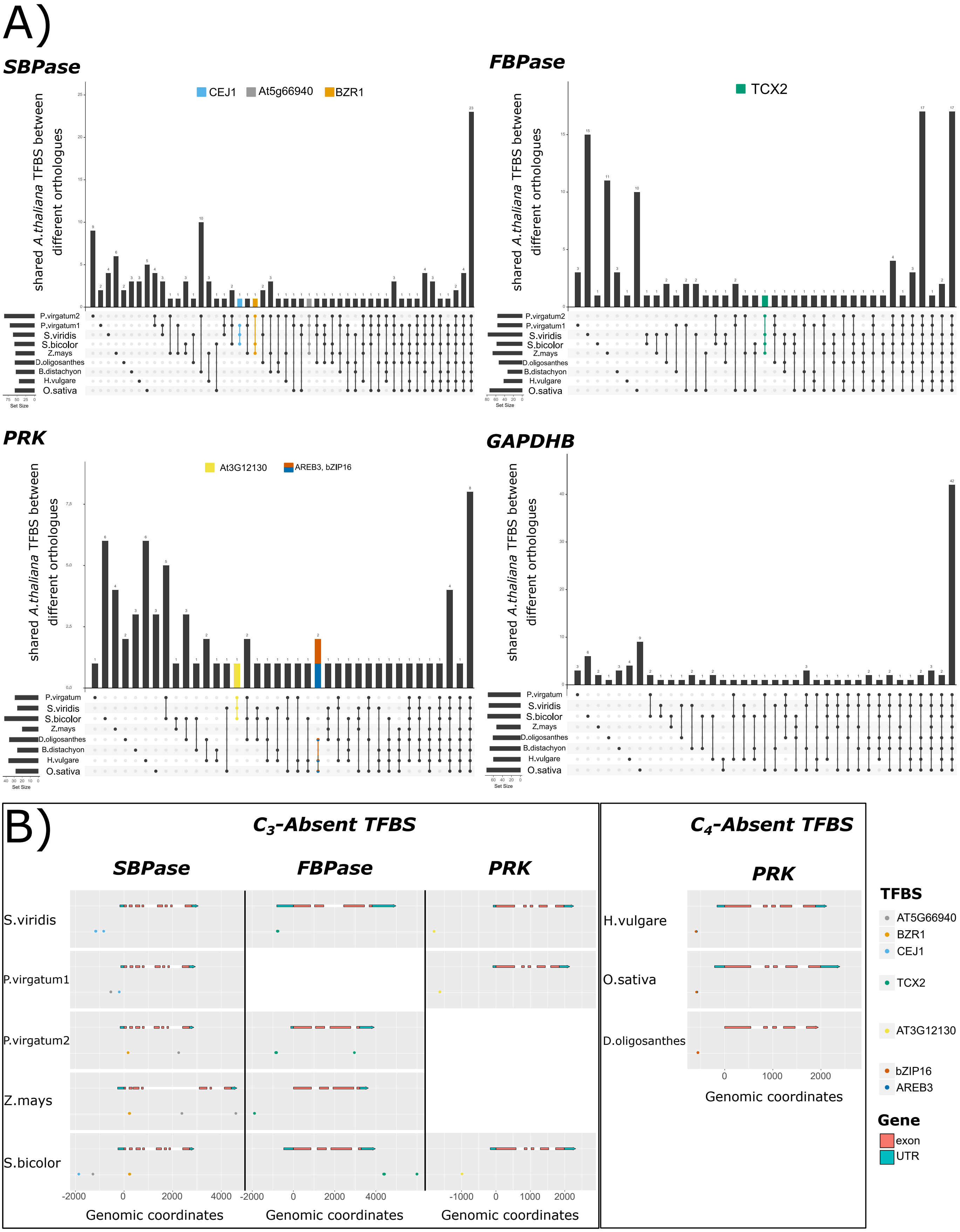

Supplementary Figure S5.- Identification of C_3- or C_4-absent motifs in the potential regulatory regions of genes encoding Calvin-Benson-Bassham cycle enzymes. A) Upset plot showing the identified *Arabidopsis thaliana* transcription factor binding sites (TFBS) and in which orthologue they are found. Horizontal bars represent the number of common motifs identified per species, vertical bars represent the motifs shared between different species, as indicated by the dots below. The number of motifs is indicated above each bar. *A.thaliana* motifs absent from C_3 species and present in at least 3 different C_4 species are highlighted. For *PRK* orthologues, we also highlighted motifs absent in C_4 and present in at least 3 C_3 species. We did not find any C_3- or C_4-absent motif in *GAPDHB* orthologues, in agreement with its high expression in both bundle sheath and mesophyll cells. **B)** Localization of C_3- and C_4-absent *A.thaliana* TFBS in the potential regulatory regions of the orthologous gene encoding the corresponding CBB enzyme. The x-axis corresponds to the genomic coordinates with the starting codon at the +1 position. The colored arrow represents the gene structure with untranslated regions (UTR) in blue and exons in red. The dots represpresent the genomic coordinates of each of the TFBS.
